# Let's talk about grief: Protocol of a study on the recognition and psychoeducation of prolonged grief disorder in outpatients with common mental disorders

**DOI:** 10.3389/fpsyt.2022.944233

**Published:** 2022-09-08

**Authors:** Simon P. N. Groen, Marijke C. Menninga, Daniëlle C. Cath, Geert E. Smid

**Affiliations:** ^1^De Evenaar Center for Transcultural Psychiatry Drenthe Mental Health Care, Beilen, Netherlands; ^2^Department of Psychiatry, University Medical Center Groningen, Rob Giel Onderzoekscentrum, Groningen, Netherlands; ^3^Drenthe Mental Health Care, Assen, Netherlands; ^4^Department of Medical Sciences, University of Groningen, Groningen, Netherlands; ^5^Department of Humanist Chaplaincy Studies, University of Humanistic Studies, Utrecht, Netherlands; ^6^ARQ National Psychotrauma Centre, Diemen, Netherlands

**Keywords:** prolonged grief disorder (PGD), prevalence, psychoeducation, post-traumatic stress, depression, refugees

## Abstract

**Background:**

Recognition that the loss of a loved one may result in prolonged grief disorder (PGD) has gained broad attention recently. PGD may disturb daily functioning to such a degree that mental health treatment is required. Because PGD symptoms often resemble symptoms of common mental disorders (CMD) such as anxiety, depressive, and post-traumatic stress disorder, clinicians may not consider a PGD diagnosis. Moreover, cultural varieties in expression of PGD may complicate recognition. This study explores the prevalence of PGD among both natives and refugees with anxiety, depressive, or trauma- and/or stressor-related disorders as well as clinicians' awareness and knowledge of PGD symptoms. In addition, a psychoeducation module on PGD symptoms is developed through patient expert collaboration.

**Methods:**

Prevalence of PGD symptoms is investigated among 50 participants who are referred to outpatient clinics for anxiety, depression, or post-traumatic stress, using the Traumatic Grief Inventory—Self Report Plus (TGI-SR+) and the Bereavement and Grief—Cultural Formulation Interview (BG-CFI). Clinicians will be interviewed on knowledge (gaps) with respect to PGD symptoms. Finally, focus groups with patient experts are held to develop a psychoeducation module tailored to the patients' needs, norms and values.

**Results:**

This study will show prevalence of PGD among patients who are referred for anxiety, depression, and post-traumatic stress, awareness and knowledge of clinicians on PGD symptoms, and will offer patient expert informed psychoeducation.

**Discussion:**

Research on prevalence and recognition of PGD is vital. Study results of the prevalence of PGD will be compared to previous studies. Recognition of PGD as distinct disorder from CMDs requires clinicians' awareness of symptoms related to the loss of a loved one. Thereby, clinicians need to take cultural aspects related to death, bereavement and mourning into consideration.

**Ethics and dissemination:**

The study protocol will be carried out in accordance with relevant guidelines and regulations. Exploratory research to assess the prevalence of PGD in patients suffering from CMDs will facilitate adequate diagnosis by increasing clinician's awareness of PGD symptoms. Tailored PGD psychoeducation, co-created by culturally diverse patient experts and clinicians will be made publicly available.

## Introduction

“To protect my family and myself from the war and violence in Syria, we moved to Egypt. Not long after we moved, the Arabic Spring started in Egypt and we were no longer safe. We then moved to Lebanon to start a new life free from violence. Unfortunately, a few days after my son got married, he died in a car accident in Lebanon. His passing turned the lives of my family upside down. We were not able to accept the loss of our son and we were sad all the time. At the same time the war situation in Syria became worse. The company that I had built up from the bottom got bombed. We then decided to flee to the Netherlands. In the Netherlands, I got the diagnosis of a post-traumatic stress disorder and a depression. Four years of treatment according to the protocols followed, by many different clinicians. I received medication and trauma treatment, but without any result. When I entered the sixth treatment with yet another clinician in a different mental health institution, he started paying attention to the loss of my son. At last I had the feeling that the core cause of my mental health problems was being addressed. The treatment of the grief over the sudden and unexpected loss of my son was confronting, but healing.”

This reflection of a Syrian refugee patient who first started to experience relief of his mental health problems after discussing the loss of his beloved son does not stand alone.

In the past 20 years, recognition that the loss of a loved one may influence psychosocial wellbeing has increased, especially if that loss is unexpected or the result of a traumatic/overwhelmingly negative experience. Following the loss of a loved one, practical, organizational, financial, and sometimes juridical issues dominate the first period. When these issues have been managed, processing the loss may start. For most people supportive contact with one or more family members, a friend, a close acquaintance, a peer, or a pastor/priest/imam/rabbi or any other religious authority, is sufficient for processing the loss. However, for some who have experienced the loss of a loved one the grief process does not get started, stagnates, or results in serious mental health problems that might require mental health care. They may develop Prolonged Grief Disorder (PGD), a disorder included in the text revision of the fifth edition of the Diagnostic and Statistical Manual of Mental Disorders (DSM-5-TR) (1) and the eleventh edition of the International Classification of Diseases (2). For clarifying purposes, the concepts of PGD according to DSM-5-TR and ICD-11 and the concept of traumatic grief are elaborated on below.

Diagnostic criteria for PGD according to DSM-5-TR (1) are summarized in Table 1. Diagnostic features according to ICD-11 further include feelings of guilt or blame about the death, inability to experience positive mood and difficulty accepting the death (2). PGD may be diagnosed after 12 months have elapsed since the death of a loved one, but the duration of normal grief reactions may vary individually and cross-culturally (1). These symptoms are distinct from bereavement-related depression and are associated with impairments in global functioning. There is an overlap between PGD symptoms and symptoms related to common mental disorders (CMDs), such as PTSD and depressive disorder. Nevertheless, PGD has a unique symptom profile. In a study among trauma-exposed bereaved adults referred for specialized treatment, PGD symptoms were common and closely associated with symptoms of PTSD and depression, highlighting the importance of assessing bereavement and PGD symptoms in patients seeking treatment following trauma (3). The term “traumatic grief” has been used to refer to prolonged grief disorder (PGD) with comorbid (symptoms of) post-traumatic stress disorder (PTSD) and/or depressive disorder following the traumatic loss of (a) loved one(s) (4). Symptoms related to both PTSD and PGD are: a traumatic event, shock, intrusive memories, and avoidance behavior related to memories of the traumatic event. Symptoms related to both depressive disorder and PGD are: grief, loss of interest, feelings of worthlessness, and feelings of guilt. Differences between PTSD and PGD include being exposed to an extreme and (potentially) life threatening situation in which fear and anxiety symptoms are the core emotion vs. being exposed to the sudden loss of a loved one, in which grief is the primary emotion. Further, in PTSD nightmares occur—as opposed to PGD—frequently. Finally, in PTSD, the person has a desire to forget events as opposed to PGD in which a person has the wish to remember the loved one.

**Table 1:** Summary of diagnostic criteria of prolonged grief disorder (1).

A. The death, at least 12 months ago, of a person who was close to the bereaved individual.
B. Since the death, the development of a persistent grief response, characterized by intense yearning for the deceased person and/or preoccupation with thoughts or memories of the deceased person.
C. Since the death, at least three of the following symptoms have been present most of the days: identity disruption, marked sense of disbelief about the death, avoidance of reminders that the person is dead, intense emotional pain, difficulty reintegrating into one's relationships and activities after the death, emotional numbness, feeling that the life is meaningless, intense loneliness as a result of the death.
D. The disturbance causes clinically significant distress or impairment in social, occupational, or other important areas of functioning.
E. The duration and severity of the bereavement reaction clearly exceed expected social, cultural, or religious norms for the individual's culture and context.
F. The symptoms are not better explained by any other mental disorder.

Meta-analytic studies indicate a pooled prevalence of 9.8% for PGD among non-psychiatric, adult populations exposed to non-violent bereavement (5), and a pooled prevalence of 49% following unnatural death and loss (6). Higher prevalence of PGD was associated with the death of a child, violent killings, and non-western study location. These findings indicate the relevance of identifying and assessing PGD in individuals exposed to bereavement and grief. Especially since most effective psychological interventions for grief-related symptoms aim specifically at grief (7), in which the reality of the loss and associated feelings play an essential role (8).

Processing grief following bereavement involved meaning attribution to loss, a process that is determined by individual, social and cultural factors as well as factors related to the circumstances of the death and the relationship with the deceased (9). Examples of sociocultural factors are norms and values related to communal acceptance of sharing emotions and cultural farewell rituals. The search for new meaning after the loss of a loved one and avoidance of thinking about the deceased play a larger role in China than in the United States (10). Norms and values related to cultural identity are considered to be particularly important, because those who have lost a loved one often report an experience of part of themselves is missing that is connecting them to their cultural background (11). In the case of refugees, bereavement and grief seem to impact their cultural identity in terms of their sense of belonging, because of the (partial) absence of a family support system, limited availability of resources to regulate the mourning process, and—although scarcely investigated—deviant norms and values with respect to bereavement and grief between refugees and native inhabitants in the host society (12), which may contribute to cultural incongruity (11). In order to improve diagnosis and treatment of PGD, increased knowledge is required on cultural differences with respect to the significance and rituals related to grief and mourning between native and refugee patient populations. One of the objectives of this study is to address this research gap.

Another research gap this study seeks to address is the awareness of PGD as a unique symptom profile among clinicians. In the diagnostic process, the focus of clinicians is often on PTSD and depression and symptoms are not always related to the loss of a loved one. Especially in the case of refugees, PGD may be overlooked, despite a high prevalence among this group (13). Awareness of PGD is crucial for adequate diagnosis and deploying psychological interventions for alleviating grief symptoms (7).

Awareness of cultural differences is also essential when providing patients with psychoeducation as part of their treatment. Adequate psychoeducation is necessary to improve patients' understanding of their symptoms and enhance effective treatment. Consulting patient experts when creating psychoeducation increases the likelihood that psychoeducation will match the patients' needs. To our knowledge, no psychoeducation on PGD has been developed with the input of culturally diverse patient experts.

The present study aims to (1) assess the prevalence of PGD in a native and refugee patient population referred for treatment of anxiety, depressive or trauma and stressor-related disorders, and, in addition, to explore differences in cultural aspects of bereavement and grief between these groups, (2) to explore awareness and knowledge of PGD among clinicians at secondary outpatient clinics of Drenthe Mental Health Care, and (3) to develop patient expert assisted psychoeducation of PGD tailored to the patients' needs.

## Methods and analyses

### Study design

**Figure 1:**
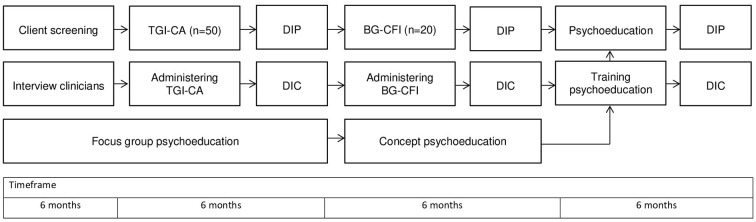
Procedure let's talk about grief study.

This study has a mixed methods design that contains qualitative as well as quantitative methods. First, presence of symptoms of PGD and CMDs in participants will be quantitatively measured. Participants with clinical and subclinical scores will be interviewed on bereavement and grief for in-depth insights into the participants' perspective on loss, bereavement and grief symptoms. Interviews with clinicians will focus on the recognition of mental health symptoms consistent with PGD. Focus groups with clinicians and patient experts on the diagnostic and treatment of PGD will be organized to develop person-centered and culturally sensitive psychoeducation. Clinicians will be trained in the newly developed psychoeducation. After they have delivered psychoeducation to their clients, debriefment for feasibility, acceptability, and clinical utility fill follow. The procedure of the study is represented in Figure 1.

### Procedure

#### Symptoms of PGD

During the timeframe of this study, patients who are referred to either a mental health unit for transcultural psychiatry in two cities (Beilen and Heerenveen) or outpatient clinics for CMDs in the city of Assen will be screened by professionals involved in the triage and/or intake on having lost (a) loved one(s) and other inclusion and exclusion criteria (Table 2). The recruitment period including assessment of PGD is planned for 12 months (Figure 1). Upon positive screening and during their first clinical appointment, patients will receive verbal and written information about this study after which a consideration period of 1 week will be taken into account. On their next clinical appointment, patients will be asked for written informed consent. Informed consent consists of voluntary participation, freedom to withdraw at any given moment during participation, and that refraining from participation has no implications for treatment. Professionals and patients involved will receive contact information to enable requesting additional information. After consent, a separate appointment will be made to assess PGD symptoms and to screen on DSM-5 classification of psychiatric disorders, carrying out (presence of DSM-5 diagnoses) the Miniscan (14, 15) and the Traumatic Grief Inventory—Clinician Administered (TGI-CA) (16, 17). A Bereavement and Grief—Cultural Formulation Interview (BG-CFI) will be administered among participants who score in the (sub)clinical range for PGD (18). To monitor feasibility, the BG-CFI will be debriefed using the Debriefing Instrument for Patients (DIP). Throughout this study, a professional interpreter will be used if necessary for communication with the patient.

**Table 2:** Inclusion and exclusion criteria.

**Inclusion criteria**	**Exclusion criteria**
Patient has lost one or more loved one(s) at or after the age of 5 years	Patient is suffering from an acute psychotic episode
Patient is at least 18 years old	Patient has cognitive impairments and/or severe substance-related disorders
Primary reason for referral of the patient is an anxiety, depressive and/or trauma- and stressor-related disorder	
The patient is mentally and physically able to fill out questionnaires and to be interviewed	

#### Mental health care professionals

After consent, 10 mental health care professionals will be asked to participate in a semi-structured interview on their knowledge, view and skills concerning PGD. The interview consists of eleven open-ended questions concerning the mental health problems of their patients, knowledge about PGD, experience with patients who have lost a loved one, experience with bereavement and grief in clinical practice, and sensitivity toward symptoms related to bereavement and grief.

#### Focus groups

During this study, 10 focus group meetings will be arranged with both native and refugee patient experts and mental health care professionals in order to discuss relevant topics concerning PGD, including norms and values with respect to bereavement and grief. All patient experts and mental health care professionals will receive information on the study and will then be asked if they are willing to participate. The focus group meetings will take place online *via* Zoom and will be recorded after consent from participants.

### Sample size

#### Focus groups

The focus groups will contain in total of six up to 10 mental health care professionals, patient representatives, and patient experts. Patient experts with and without a migratory background will be recruited from formal patient expert education. Patient representatives are refugees with experience in mental health research who have not been a patient themselves. The inclusion of patient experts aims to focus on the patients' needs, norms and values from their own experiences.

#### Prevalence of PGD

Fifty patients are needed to include for the descriptive statistical analysis. We aim to include 25 participants in the refugee and 25 participants in the native group. Based on prevalence numbers of PGD in several studies, we aim to include fifty patients to meet the required number of twenty respondents who score above threshold levels for PGD according to DSM-5-TR and ICD-11 on the TGI-CA. The number of twenty patients is estimated as feasible, while valid statements can still be made on the basis of the results. Only descriptive and exploratory comparative statistical analyses will be carried out. Participants will be recruited from outpatient clinics patients within Drenthe Mental Health Care, department of transcultural psychiatry and the outpatient clinics for CMDs. We deliberately include both native and refugee patients in equal proportions in this research in order to establish a diverse study population. A culturally diverse study population enables generalization to a variety of patients when it comes to recognizing PGD and providing psychoeducation. In addition, a culturally diverse study population enables us to make a comparison between native and refugee patients. Inclusion and exclusion criteria are listed in Table 2.

### Measures

#### PGD

To assess PGD, the Traumatic Grief Inventory—Clinician Administered (TGI-CA) will be employed among participants (17). The TGI-CA is a validated questionnaire with 22 items on a 5-point Likert-scale (1 = never, 5 = always). The TGI-CA is available in English, Dutch and German. If the language of the participant is not available and/or the participant is not able to read, an official interpreter will be used. The psychometric validation of the TGI-CA is currently carried out (17), but the validation scores of the Traumatic Grief Inventory—Self Report Plus (TGI-SR+) show strong internal consistency and strong correlations with psychopathology and a low quality of life (16). A score of 71 or higher is an indication for the presence of PGD (16).

#### Miniscan

To assess the prevalence of psychological symptoms as listed in the DSM-5, the Miniscan will be applied. The Miniscan is a shorter version of the Schedules for Clinical Assessment in Neuropsychiatry (SCAN) of the World Health Organization (15). It's a web- and interviewer-based semi-structured interview that can be used to assess psychological symptoms of Axis 1 disorders according to DSM-5. Psychometric properties of the Miniscan were determined: when compared with the SCAN, substantial agreement was found with a Cohen's kappa for concurrent validity of 0.802 (s.e. = 0.045). Sensitivity, specificity, positive and negative predictive value and efficiency were all very good, with the exception of the sensitivity and predictive value of affective psychoses that were still substantial (15). For this study, only the segments on anxiety, depressive, and trauma-or stressor-related disorders will be used. The goal of using the Miniscan is to be able to differentiate between these disorders.

#### Perspective of patients on loss, bereavement and grief symptoms

The Bereavement and Grief Cultural Formulation Interview (BG-CFI) will be used to explore the context and the perspective of patients on loss, bereavement and grief symptoms. The interview will be conducted by researchers with participants who have a (sub)clinical score on the TGI-CA. The interview is available in English and Dutch (19, 20). If necessary, a professional interpreter of an official Dutch translation service will be used. The BG-CFI is a semi structured interview that consists of 18 open questions, divided into two themes: cultural traditions around dead, loss and grief and help seeking behavior and coping with loss of loves ones (19). Questions are, for example, about ways of mourning and rituals around loss that are important to relatives.

#### Development of psychoeducation

The development of psychoeducation about PGD will be facilitated in 10 focus group meetings with mental health care professionals and patient experts. The meetings of this focus group will take place online. Each meeting will have one central theme. Themes that will be discussed are, for example: what should we be aware of when talking about grief? What can be discussed and what can not? What to do when a patient is hesitant to talk about grief? Attention will be paid to adapting psychoeducation to the knowledge level of patients, using language that is adapted to patient's understanding, being aware of communication barriers and subsequently discussing and solving them, and to a connection between a theoretical orientations and practical applications.

#### Evaluation of BG-CFI and psychoeducation

The BG-CFI and patient expert informed psychoeducation will be evaluated through an adapted version of the Debriefing Instrument for Patients (DIP). This instrument has previously been used in research on the Cultural Formulation Interview, which is included in the DSM-5 (21). The DIP will be adjusted to be used for evaluating the BG-CFI and the psychoeducation on PGD. The DIP consists of eight questions about the clinical utility, three questions about the feasibility and three questions about the acceptability. It has a five-points Likert-scale (1 = strongly disagree, 4 = strongly agree, 5 = not applicable). The DIP also contains open questions, such as “What additional questions would have helped your clinician understand your concerns better?” The DIP is available in several languages. If necessary, an official interpreter of an official Dutch translation service will be used. Clinician's experiences of the BG-CFI and psychoeducation will be evaluated using an adapted version of the Debriefing Instrument for Clinicians (DIC) that was used in the international CFI field trial (21). The DIC has a similar design as the DIP; it consists of 10 items about clinical utility, four items about feasibility and four items about the acceptability, with the same five-point Likert scale.

### Data analysis

#### Qualitative data

Reports of the focus group meetings, the BG-CFIs and open-ended questions of the DIP and DIC will be qualitatively analyzed on most relevant themes that need to be addressed in the development of psychoeducation. Computer-assisted qualitative data analysis software Atlas.ti 8.0 will be used for in-depth qualitative thematic analysis (22). Atlas.ti is based on a grounded theory approach, that inductively builds theory from empirical data (23). Reports from focus group meetings will be thematically analyzed with consideration for key ingredients for psychoeducation related to grief and bereavement.

Themes of the focus group meetings will concern experiences with grief and bereavement; communication between patient and clinician; required information; barriers for reprocessing; the role of family, loved ones and others; and attitude of clinicians during treatment. Retrieved information will be thematically analyzed and reviewed in the focus group by means of member check to raise credibility (24). Patient expert informed key ingredients for bereavement and grief psychoeducation will be collected into a test version. Mental health professionals will administer and evaluate the test version of grief and bereavement psychoeducation. Qualitative results from the patient debriefing of the BG-CFI will be included in the psychoeducation. Qualitative results from the clinician debriefing of the BG-CFI will be included in the psychoeducation training of clinicians. The COREQ checklist will be applied for explicit and comprehensive reporting of the study (25).

#### Quantitative data

Results from the Miniscan and the TGI-CA will be used to answer the research question that concerns the prevalence of PGD according to DSM-5-TR and ICD-11 in the research population and comorbid diagnoses. Descriptive analysis will focus on comorbidity of PGD with PTSD, depression, and generalized anxiety disorder. Exploratory comparative group analyses will focus on prevalence of PGD according to DSM-5-TR and ICD-11 and comorbidity of PGD with PTSD, depression, and generalized anxiety disorder.

## Discussion

Recent developments in the classification of mental disorders have resulted in the recognition of symptoms that are related to PGD. Recognition of PGD in patients who suffer from CMDs is both relevant and complicated because of symptom overlap between PGD, PTSD, and depression. Clinicians may classify CMDs with neglect of the potential relation of these disorders with the loss of a patient's loved one. In clinical practice, distinctions between symptoms related to PTSD or depression may not be clear to the clinician if the question whether there is loss of a loved one is left unasked. The clinical case example in the introduction shows that failed or late recognition of PGD may result in inadequate treatment and unsuccessful response.

Although cultural variations in response to bereavement and grief such as cultural traditions related to death, bereavement, and mourning may be omnipresent, cultural aspects of PGD are under researched. To facilitate clinical exploration of those cultural aspects, a Bereavement and Grief Cultural Formulation Interview (BG-CFI) has been proposed (19). Feasibility, acceptability, and clinical utility of the BG-CFI have yet to be tested. In this study, assessment of the BG-CFI will be debriefed using adapted versions of the debriefing instruments that have been used in the international field trial of the Cultural Formulation Interview (CFI) (21). The CFI aims to elicit cultural aspects of mental health problems in every patient, because there may be cultural variations in every individual. In this study, native patients will be assessed with a CFI as well. Local norms and values of bereavement and grief will include community aspects such as various attitudes toward death, bereavement and mourning between generations, closed and open communities, and various religious backgrounds.

Strengths of this study are that both CMDs and PGD will be assessed allowing diffusion in diagnosis to be elicited, that cultural variations related to death, bereavement, and mourning will be included, and the involvement of (native and refugee) patient experts in the development of psychoeducation. Limitations of the study are the potential influence of implementing the study on the awareness of PGD among clinicians, and stigma-related, attitudinal, or instrumental barriers to participate in the study.

## Ethics and dissemination

The study protocol has been submitted for ethical approval at the University Medical Center Groningen (UMCG, 2021/398). The Medical-Ethical Review Commission decided that ethical reconsideration was not required as a clinical study. However, the study protocol was then submitted for ethical approval of the Psychology Department, University of Groningen (PSY-2021-5-0527) and approved. Exploratory research to assess the prevalence of PGD in patients suffering from CMDs is necessary, because of the potential risk that clinicians tend to classify presented symptoms without consideration of the relation with the loss of a loved one. A lack of such consideration may lead to classification of anxiety, depressive, or trauma and stressor-related disorders, while symptoms may be related to PGD. PGD requires different types of treatment, or a focus in treatment on the relation between the symptoms and bereavement and grief. The case study example clearly shows that four types of trauma-focused treatment did not result in symptom reduction, whereas treatment focused on grief and bereavement did. Therefore, the main ethical aim of this study is that patients suffering from PGD obtain accurate diagnosis and treatment.

Another ethical aspect of this study is the inclusion of patient experts in the development of psychoeducation on PGD. Often psychoeducation has been developed top-down without patient consultation. Such development may result in ineffective psychoeducation that insufficiently connects to the patient's needs. Therefore, focus group methods are applied in this study including patient experts to represent patient perspectives in the development of psychoeducation on PGD. Moreover, due to increasing cultural diversity of patient populations, the inclusion of refugee patients, and to engage into present-day diversity and inclusion requirements, this study focusses on patient's norms and values as well. Therefore, the selection procedure for members of the focus group will include cultural diversity preferences. Patient-informed focus group meetings will be applied to gather most important themes of PGD, the patient's needs, and norms and values related to grief and bereavement. Gathered information will be analyzed using computer-aided qualitative analysis. The analysis will be peer debriefed in focus group meetings and applied in psychoeducation folders. The study results will be presented at relevant national and international symposia.

## Author contributions

SG is the primary investigator and has drafted the manuscript. MM, DC, and GS have revised and approved the final version of the manuscript. All authors contributed to the article and approved the submitted version.

## Conflict of interest

The authors declare that the research was conducted in the absence of any commercial or financial relationships that could be construed as a potential conflict of interest.

## Publisher's note

All claims expressed in this article are solely those of the authors and do not necessarily represent those of their affiliated organizations, or those of the publisher, the editors and the reviewers. Any product that may be evaluated in this article, or claim that may be made by its manufacturer, is not guaranteed or endorsed by the publisher.
